# Forecasting migration movements using prediction markets

**DOI:** 10.1186/s40878-024-00404-0

**Published:** 2024-10-09

**Authors:** Sandra Morgenstern, Oliver Strijbis

**Affiliations:** 1 Mannheim Centre for European Social Research (MZES), University of Mannheim, Mannheim, Germany; 2 Franklin University Switzerland, Sorengo, Switzerland; 3 Institute of Political Science, University of Zurich, Zurich, Switzerland

**Keywords:** Migration forecast, Mobilities, Methodological advancement, West European countries

## Abstract

Migration forecasts are crucial for proactive immigration and integration management. While the demand for accurate migration forecasts continues to grow, the current state of migration forecasting is still unsatisfactory, because they tend to lack precision. We introduce an alternative method to forecast migration movements: prediction markets. While prediction markets are mainly unknown in migration studies, they are established in the political economy of forecasting election outcomes. For its application to a complex phenomenon in a more constrained information environment such as migration movements, we argue that prediction markets allow to balance complementarities of current qualitative and quantitative approaches if they provide solutions to avoid thin trading and integrate expert knowledge into the market. We apply the prediction market to forecast immigration in four West European countries in 2020 and find encouraging results. We discuss the strengths and limitations of prediction markets to migration forecasting, including ethical considerations, and guide its future application.

**Supplementary Information:**

The online version contains supplementary material available at 10.1186/s40878-024-00404-0.

## Introduction

The high numbers in migration movements around the year 2015 were an unexpected challenge for European destination countries. Governments, non-governmental organizations (NGOs), but also the broader society were not sufficiently prepared; not for the short-term concerns such as accommodation and neither the long-term consequences such as extension of the public infrastructure and the provision of integration courses. Among other reasons, this was the case because none of the forecasting models had predicted this increase in immigration at that time (Sohst et al., 2020). This lack of preparation had severe implications for the labor and housing market, public transport, economic growth, retirement provisions, health care, and public schools, to name but a few examples. Indeed, the rapid increase in migration in 2015 is a particular case. However, as the current rise in migration from countries at war in and around Europe reminds us, quickly accelerating and strong migration movements repeatedly recur following extraordinary important political and economic events that until now were not reflected sufficiently in forecasts.

To avoid or at least minimize this reactive way of migration management and prepare for migration movements, it is vital to forecast migration dynamics as accurately as possible (Anderson, 2017; Castles, 2004). However, although the need is clear and the demand exists (Bijak et al. 2017; Kjærum, 2020), the current state of the art on migration forecasting is still unsatisfactory (Disney et al., 2015; Sardoschau, 2020; Sohst et al., 2020). Main reasons are the challenges that come with forecasting in general (Bijak, 2010). However, given the high complexity of migration and the inherent information constraints, the challenges are further amplified when forecasting migration movements (Bijak & Czaika, 2020; Willekens, 1994).

With this article, we introduce and assess a method not yet applied in migration research that might allow for accurate and rapidly updated forecasts: prediction markets. Prediction markets, also known as ‘information markets’ or ‘betting markets,’ are virtual markets that work like financial markets. In contrast to financial markets, the market participants do not trade financial assets but expectations on all kinds of future events. As distinguished scholars in economics argue, prediction markets are better able to predict a large variety of events than traditional prediction instruments (Arrow et al., 2008). Prediction markets have been shown to be accurate in forecasting election outcomes and foresee highly complex events such as the success of replication studies in social and behavioral science (Gordon et al., 2021). Nonetheless, prediction markets have not been used yet to predict migration movements.^1^

In this article, we discuss the specifics of an appropriate prediction market design for forecasting complex questions in a context of a restricted information environment on an ethically challenging topic. Thereby we build on the efficiency of the price signal in aggregating decentralized information and the independence of the participants with regard to their expectation judgements avoiding groupthink processes. Based on the setting, we argue for a probability market applied to a sample consisting of a substantial number of laypeople and a selected panel of experts. We apply our market design to the forecasting of immigration and asylum applications in four European countries. The results show that the prediction market might have the potential to improve the forecasting of migration movements. However, we also discuss its limitations and potential avenues for future application and assessment of this method.

## State of the art: methodologies for forecasting migration movements

For most social scientists, engaging in predictions is an exercise in theory testing linked to the Popperian epistemology of critical rationalism. From this perspective, the main merit of predicting—be it the past or future, quantitatively or qualitatively—is to put explanations to a critical test in order to revise them. The relevance of forecasting, in contrast, is not in theory testing but in informing decision making (Tetlock and Gardner 2015). Forecasts are helpful if they predict the future as accurately as possible. They are accurate when they are good in discrimination, i.e. give specific events probabilities that are close to 0% or 100%, and are well calibrated, i.e. assign types of events the probability of happening which they do on average (Tetlock, 2009). Since arriving at accurate forecasts and theory testing are two different aims, the fundamental logic of each can be distinct. For instance, while theory can be helpful in forecasting migration movements (Willikens 1994) “the nomothetic forecasting of migration, based directly on the laws or theories of population movements, is not an option, as the existing laws and theories are not universal enough to allow for the practical application of this approach” (Bijak, 2011, 51). To summarize, accurate forecasts are often based on some form of extrapolation rather than on explanatory theory, while explanatory theory is central to hypothesis testing.

Multiple concepts with varying definitions are used in the literature of migration forecasting (Bijak et al. 2017; Sardoschau, 2020; Sohst et al., 2020). In this article we use the term ‘forecast’ to refer to short-term forecasts into the future. This is in contrast to long-term forecasts, which are known as ‘foresights’, immediate forecasts, which are known as ‘nowcasts’, and ‘predictions’, which can cover both future and past directions. In migration forecasting, we can distinguish between three overarching methodological approaches. In the following, we will elaborate on major streams within each of the three and set them into perspective regarding their methodological advantages and disadvantages. However, this overview is by no means comprehensive. For more comprehensive literature reviews about methods forecasting migration, see inter alia Sardoschau (2020) Sohst et al. (2020), and Bijak (2010).

First, qualitative methodological approaches, which typically rely on insights from a limited group of participants, generally experts. The main strength of these approaches is that they are not necessarily dependent on migration statistics and certain model assumptions. The most prominent approach is the so-called ‘migration scenario creation process,’ which—when applied to broad questions of “what will happen”—builds various general migration forecasts based on expert discussions and a theoretically deduced identification of migration drivers (De Haas et al., 2010; Vezzoli et al., 2017). Besides the difficulty to theoretically identify key drivers based on a complex and fragmented spectrum of migration theories (Bijak & Czaika, 2020), a limitation is that the forecasts may vary strongly depending on the experts that are sampled and/or might be biased through the impact of opinion leaders.

Second, quantitative methodological approaches that avoid the strong dependence on expert opinions through the systematic analysis of large numbers of data points and further reduce subjectivity using official migration statistics. Yet, even in quantitative forecasting subjectivity is inevitable, as the researcher makes model assumptions. Structural Equation Models (SEM) rely especially heavily on theoretical assumptions, and SEM international migration predictions come accordingly with high uncertainties (Burzynski et al., 2022; Dao et al., 2018). Gravity models are less theory-dependent than SEMs but must rely on almost time-invariant variables to make good migration forecasts, making them relatively rigid (Cohen et al., 2008; Hanson & McIntosh, 2016). Bayesian statistical modeling became the preferred technique because it allows to include the uncertainties introduced through theoretical assumptions into the model and—as shown for forecasts on asylum migration—only depends on some data distribution assumptions (Azose & Raftery, 2019). The most recently developed models use machine learning models applied to the forecasting of asylum migration (Carammia et al. 2020; Robinson & Dilkina, 2017).

Beyond firm reliance on modelling assumptions, quantitative forecasting models face a special challenge when forecasting migration as suitable data is often not available—the core source of these atheoretical models rise and fall with the quality of the data in their data-driven approach. Migration data is generally based on administrative statistics. The variety of data collection practices across registration offices within and between countries hampers comparability, censuses are only implemented in longer time intervals (and interpolated for the interim years), while the inherently different purpose of data collection^2^ leaves us with rather poor data quality regarding migration. This, in turn, leads to high uncertainties in the forecasts (Abel et al., 2013; Raymer et al., 2013). The limited quality and availability of data are mainly challenging for the popular Bayesian and machine learning approaches since their usefulness depends heavily on the availability of large volumes of data that cover long time–series. Data limitations also prevent forecasting migration movements for different migration categories (Sohst et al., 2020). Newer approaches, that are often in the realm of ‘nowcasts’ compensate for poor data quality from official statistical records by using less traditional sources such as Facebook (Palotti et al., 2020; Spyratos et al., 2018) or Google search activities (Böhme et al. 2020; Wanner, 2020) to forecast migration patterns. However, the improvements in the data collections come with new shortcomings regarding lacking representativity, not at least due to low internet coverage in key origin countries of interest outside the global north (Carammia et al. 2020; Rampazzo et al. 2024).

Third, probably the most promising models to accurately forecast migration movements are based on mixed-method approaches. The different kinds of uncertainties resulting from qualitative and quantitative approaches and their respective model strengths greatly complement each other. For example, Acostamadiedo et al. (2020) use qualitative migration scenarios for the guidance of the quantitative expert questionnaire. Billari et al. (2014) proceed vice versa—i.e. they apply first a method from the qualitative toolkit and secondly a quantitative method—by using a quantitative model on top of expert projections. Although both procedures—in either chronological order—may outperform prior models, they are still limited in their forecasting accuracy.

Comparisons of current attempts in migration forecasting with regards to their accuracy are difficult and consequently rare (Casagran et al. 2021; Sardoschau, 2020). Moreover, due to funding constraints, forecasts are often not continued over time but only for specific time points. Continuously updating modeling systems as recommended by Bijak et al. (2017) would be necessary for allowing comparisons and forecast short-term trends like the so-called migration crisis in 2015.

To summarize, both quantitative and qualitative models of migration forecasting suffer from severe methodological or practical limitations. Consequently, mixed-method approaches aiming at exploiting the complementarities of qualitative and quantitative techniques seem most promising. In the following, we will describe prediction markets as a methodology that falls into the category of mixed methods. By letting a considerable number of laypeople and a smaller sample of experts reveal their expectations on a prediction market, prediction markets limit the dependency on the expert judgment through the wisdom of crowds without entirely erasing expert knowledge as a source of the forecasts. Furthermore, the prediction market method avoids the challenge of quantitative modeling assumptions based on the complex and manifold migration theories. Finally, in the prediction market, a continuous updating based on new information is possible, and hence the method could be beneficial for its sensitivity to short-term trends. However, we do not claim that prediction markets are a method that may solve all the above raised difficulties of migration forecasting and we will reflect on the necessary assumptions and limitations in the Conclusion.

## A new methodological application: prediction market on migration movements

Prediction markets, as defined by Berg et al. (2008), are Internet-based financial markets designed to use the information contained in market prices to make predictions about certain future events. With the aim of bringing to light the best collective assumptions, the participants buy and sell (henceforth ‘trade’) their expectations regarding specific events, called ‘contracts,’ that will occur in the future. The values of traded contracts depend directly on future events, and therefore the prices of these contracts provide information about the events. Applied to our case this means that participants trade their expectations about, for example, the number of immigrants to a specific country in a certain year and the researcher or market administrator may take the prices—a result from the trading based on perceptions—as the prediction information.

Prediction markets belong to the category of ‘aggregators’ in that they aggregate individual human forecasts. As such they are atheoretical because they aggregate forecasts independent of whether they are based on any theoretical assumptions. This does, of course, not mean that migration theory is fully absent. Given that the market is an aggregate perspective of multiple individual forecasts, we assume that migration theories enter indirectly in this stage. The degree to which migration theory is applied depends on the participant. To make valid claims about the inclusion of migration theories and participants’ assumptions, future investigations will need to combine the market with a qualitative investigation of mind processes among the participants.

The backbone of prediction markets (as for any market) is the efficient market hypothesis (EMH), which states that prices reflect all information (Fama, 1970; Hayek, 1945). We do not assume that this assumption perfectly holds on our market on migration, nor any other market. Markets tend towards information inefficiencies (Grossman & Stiglitz, 1980) and potential ‘misvaluation’ as a consequence of limited rationality among individuals (Hirshleifer, 2001; Kahneman & Tversky, 1973).^3^ We *do* assume, however, that the price mechanism is efficient enough in aggregating even highly decentralized information and is consequently superior to most other aggregation mechanisms such as the calculation of some type of average (e.g. mean, median). Also, we do assume that the fact that participants on prediction markets arrive at their expectations independently reduces systematic bias through avoidance of “groupthink” and “opinion leaders”, which are the weaknesses of aggregators that allow for deliberation such as focus groups.

The assumption of independent information may seem counterintuitive at first. Participants in the prediction market—laypeople and experts—live in the same world, and with the increasing availability of information in a globalised and digitalised world, people may have access to the same sources of information and ergo the same information. However, there are two relevant reasons why, even in such a situation, individual information intake may lead to the aggregation of diverse information: First, there is a selective search mechanism. As shown in the literature on filter bubbles and selective exposure (e.g. Flaxman et al., 2016), people consume different information. Second, even in settings with the same information intake, dual-process theories from the psychological literature suggest that information processing is individual and leads to different levels of available information in a decision-making setting (e.g. Morgenstern, 2023). We would expect the variability to hold for both groups, but to be much smaller for experts than for laypeople. We still expect participants to have sufficient information, as migration is a highly salient topic in recent years, although not as much as election outcomes—the case where prediction markets have primarily been applied so far.

If the prediction market aims to forecast the likelihood of an event, a probability market needs to be applied. In probability markets, the final values of the contracts are defined as 100 if the event materializes and 0 else. Assuming risk neutral utility maximizers,^4^ this translates into probabilities. The reason is that these economically rational participants trade shares based on the probabilities they attach to events. For example, suppose a rational trader on the market thinks that the likelihood of an outcome is 60% while the share’s current price is at 50. In that case, she buys as many shares until the price is at 60 because—until this value—the expected value (EV) is above 0.^5^

While the probability market is the proper setup to forecast the likelihood according to which events occur, for some questions, we are not only interested in the likelihood of an event. Instead, we are also interested in the specific point estimate. For example, we might want to have a point estimate for the number of immigrants that will arrive in a given year. One option to arrive at such point estimates is defining the final value according to the specific value (e.g., 0.5 if the outcome is 500,000). This type of market, sometimes called ‘index market’, can directly generate point estimates (Snowberg, Wolfers, and Zitzewitz 2013). It has the disadvantage that it does not come with information on the uncertainty of the forecast and is particularly prone to bias introduced by market scoring rules (Arnesen & Strijbis, 2015; Strijbis & Arnesen, 2019).

In order to arrive at point estimates with uncertainty levels, the so-called ‘sequencing method’ (Strijbis, 2020) can be applied. This method provides tradable contracts for different mutually exclusive ranges (e.g., ranges of the number of immigrants) and interpolates the probabilities attached to each.^6^ The interpolated outcome (e.g., number of immigrants) that lies at the 50% probability level is the point estimate since the probability of the outcome being lower or higher is smaller in both cases. In addition, the resulting probability distribution can be used to arrive at probability intervals that measure the uncertainty according to which the outcome will fall into a specific range. The only major disadvantage of this approach is that the number of ranges offered as contracts for trading has to be somewhat limited to avoid market inefficiency. Hence, it must be assumed that the cumulative distribution function (CDF) created by interpolation approximates the true probability function that underlies the trading transactions of the participants on the market.

Prediction markets have proven themselves in practice in particular in the forecasting of elections in the USA (Berg et al., 2003, 2008). They have been widely used in Europe, where on average they have outperformed other forecasting models based on polls, expert panels, and economic indicators (Graefe, 2017).^7^ While prediction markets generally allow for accurate forecasts, forecasting accuracy is not constant but can vary in important ways even for the same events (Strijbis & Arnesen, 2019). A much-discussed problem of prediction markets is ‘thin trading’ (Pennock & Sami 2007). Thin trading results from a lack of matching buy and sell offers. Applied to our case, the situation would occur if the participant preferred a certain trade given her expectations on migration movements but doesn’t get the opportunity to follow her plans due to the market situation. As a result, no market price is generated, and therefore, no forecast can be derived. Large samples of participants can prevent the probability for thin trading. However, automated price makers may ensure infinite liquidity (Hanson, 2003, 2007; Othman et al., 2013) using an algorithm that offers a new price for the expectations on migration after each transaction and prevents a situation where the participant is not willing to accept the price. The most popular market scoring rule—which we also implement in our application (see below)—is the Logarithmic Market Scoring Rule (LMSR) developed by Hanson (2003) as it is known for least systemic bias introduction in forecasts (Dudík et al., 2017).^8^

Predition markets rely not only on efficient market theory, but also on the ‘wisdom of crowds’ literature, which indicates that larger samples reduce prediction errors (Galton, 1907; Surowiecki, 2005). Dudík et al. (2017) also show that this is also true for prediction markets with market scoring rules where under certain conditions the discrepancy between market clearing prices and ground truth goes to zero as the population of traders increases. However, how much the wisdom of crowds mechanism plays in prediction markets and how many participants need to participate in a prediction market to arrive at accurate forecasts is still an unresolved issue. While the wisdom of the crowd theory suggests that the number of participants should be considerable, some argue that market efficiency is achieved already with a handful of participants. For instance, Christiansen (2007) reported in a case study that prediction markets with more than 16 participants were well-calibrated and McHugh and Jackson (2012) found varying the number of participants in the prediction market has a minimal impact on its accuracy.

Most likely, whether prediction markets are efficient with few participants depends heavily on whether the participants have access to relevant information. Therefore, especially when the relevant information is not readily available or hard to process for laypeople, expert panels are often the choice. The information these experts bring into the market should be seen as complementary to the wisdom of crowds generated through the participation of many laypeople.

To summarize, making sure that there are enough participants with access to relevant information there are no reasons why prediction markets should not also be able to aggregate the available information on migration movements in a highly efficient way and hence to generate accurate migration forecasts. This is also what prediction markets that have successfully been applied to forecast the success of replication studies in social and behavioral science (Gordon et al., 2021)—outcomes that are particularly hard to forecast—suggest.

## Ethical considerations

Since migration and especially asylum migration is a sensitive topic, it is necessary to reflect upon the ethical dimension of each step of a new methodological approach applied to the topic. Before design and implementation, we reflected upon the intentions behind the study and its potential impact on society. This helped to set activities into relation and a proceeding of the research project in the first place. Here, the intentions are to improve current forecasting about migration movements and asylum applications to switch from reactive to proactive migration management to ease the arrival of migrants.

The aim of improving the arrival of the migrants and the participants recruited for the prediction market—migration experts and trained laypeople—implies that the primary beneficiaries do not match the sample and hence the investing group. A mismatch like this is a relevant point considering the invasiveness of the study. For the expert sample, we do not expect significant invasiveness of the study and the participation. However, the laypeople could be influenced negatively in the sense that applying the stock market view of the prediction market to the movement of actual people might change their perception of migrants and make them ‘less real.’ To minimize the potential for such an outcome, we briefed all participants in advance and debriefed them in the aftermath of the study.

A dilemma in designing the study was the decision regarding the incentivization of the study participants. On the one hand, research regarding prediction markets encourages a monetary incentive due to its monetary logic allowing the markets to work correctly (Luckner & Weinhardt, 2007; Rosenbloom & Notz, 2006; Servan-Schreiber et al., 2004). On the other hand, the financial incentives might also motivate participants to influence the study subject—migration movements—in the real world. For the laypeople sample influencing actual migration seems to be an impossible task and is not risky. In contrast, the experts could influence migration movements since they are, by definition, people sampled from institutions that have to different degrees an impact on either immigration or integration practices. To avoid losing the monetary logic of the market while preventing a push to influence real-life migration for own monetary gains, we decided to donate the ‘gains’ from the market to a humanitarian organization of the participant’s choice. We informed them accordingly in advance of the study and participants could choose the organization out of some preselected public good oriented NGOs in the aftermath. We do not have any role models in this regard for prediction markets, but reflecting on the mechanism of the financial logic it might be the case that monetary incentives for the individual might trigger egoists and capitalists slightly more, while donations trigger stronger on altruists. However, there is no reason to assume that these different levels of trigger will have a substantial impact on the performance in the market.

## Application of the method: forecasting migration movements in 2020

In the following, we apply the described prediction market to the forecasting of migration movements in 2020 (see the Supplemental Material part A and B). More precisely, we focus on asylum applications and immigration in general (i.e., we do not distinguish between labor migration, education migration, and family reunification migration). The overall application setup was as follows: In May 2020, we opened the prediction market and invited the target sample of potential participants. Reminders to update their ‘portfolios’ according to their expectations were sent to the participants in July and November 2020. The prediction market was closed with the end of the year and hence the date for which the forecast was made. In this article, we evaluate the forecasts as of May 31, 2020—the first time point at which a majority of the participants were enrolled in the market.

Prediction markets generate forecasts for specific events. As long as the time horizon is limited, these events can be of almost any kind. However, events must be clearly defined, and hence it is vital that at a later stage, hard data exists that allows judging whether a specific event has taken place. This is the case for events for which statistical offices provide data. The number of events that can be used is also not limited as such. However, a prediction market with too many questions on which participants can trade markets can lead to thin trading since the participants might concentrate on only a few of them.

We limited the prediction market to migration movements to four destination countries and three topics in 2020. The destination countries comprise Germany, Spain, Switzerland, and the UK. The first two being continuing EU countries, Germany known as a classic immigration country, and Spain a (still) newer immigration country. Switzerland represents a non-EU, but Schengen case and the UK a country in transition from EU to a non-EU country outside the Schengen area. The topics of the markets are for each country the number of foreign immigrations in 2020 and the number of first-time asylum applications in 2020. (In Supplemental Material part F we also report and evaluate forecasts for the most frequent country of origin among first-time asylum applicants in 2020.) We excluded immigration to the UK because we were unsure if the migration statistics to settle the market would be published within a reasonable time horizon and could rely only on few British participants. For a detailed description of the data sources and historical data please see the Supplemental Material part C.

Since we predicted migration movements for the entire 2020 during the same year, our predictions were only partly forecasts for the future and to some extent estimations for past migration movements in the absence of official accounts, i.e. so called “nowcasts”.^9^ We decided to forecast migration statistics for the ongoing year for two reasons. First, only for 2020 (rather than 2021) would we have meaningful data to settle the market in due time. Indeed, for some of our forecasting questions official accounts were not available for the 2019 until late in 2020. Hence, by forecasting migration movements in 2021, we would have needed to wait to evaluate our forecasts until the second halve of 2022 at least. This would have been unpleasant for the participants since they would have had to wait more than one year to learn how well they did. With our design, instead, they ‘only’ had to wait halve a year since in June 2021 the official statistics became publicly available for all markets, which allowed to settle the markets and calculate the final score for each participant.

In order to evaluate the accuracy of the prediction market forecasts, we will compare them with forecasts that we have generated ex-post based on standard time series models. Due to the low number of cases, we will discuss the accuracy qualitatively by comparing each point estimate with the outcome and the alternative forecasts from the time series models. Additionally, we will rely on standard measures based on the mean average error (MAE).

### The participants

When forecasting migration movements relevant information is not readily available and hard to process given the complexity of the migration topic. Consequently, in order to combine the logic of the wisdom of crowds and benefit from the experts’ knowledge, we created a panel consisting of both experts and laypeople (see above). Additionally to relying on a pool of participants that participated in prior prediction markets on elections, we actively recruited experts to participate in our market.^10^ Experts may be defined as migration researchers being firm in the theoretical models and assumptions on migration, administrative officers working on the judicative aspect of migration, or members of NGOs and IGOs working on the ground with exceptional practical knowledge. According to the logic of a decentralized aggregation mechanism of markets, these experts ideally have diverse information. To guarantee diversity, we invited migration researchers, experts from NGOs, and governmental organizations to participate.

**Fig. 1:**
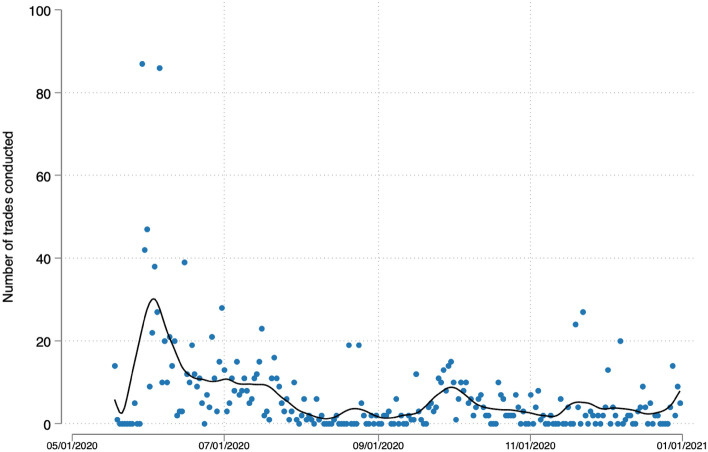
Number of trades conducted. The dots show the number of trades conducted for each day with the line showing the respective LOESS function with bandwith = 0.1

**Fig. 2:**
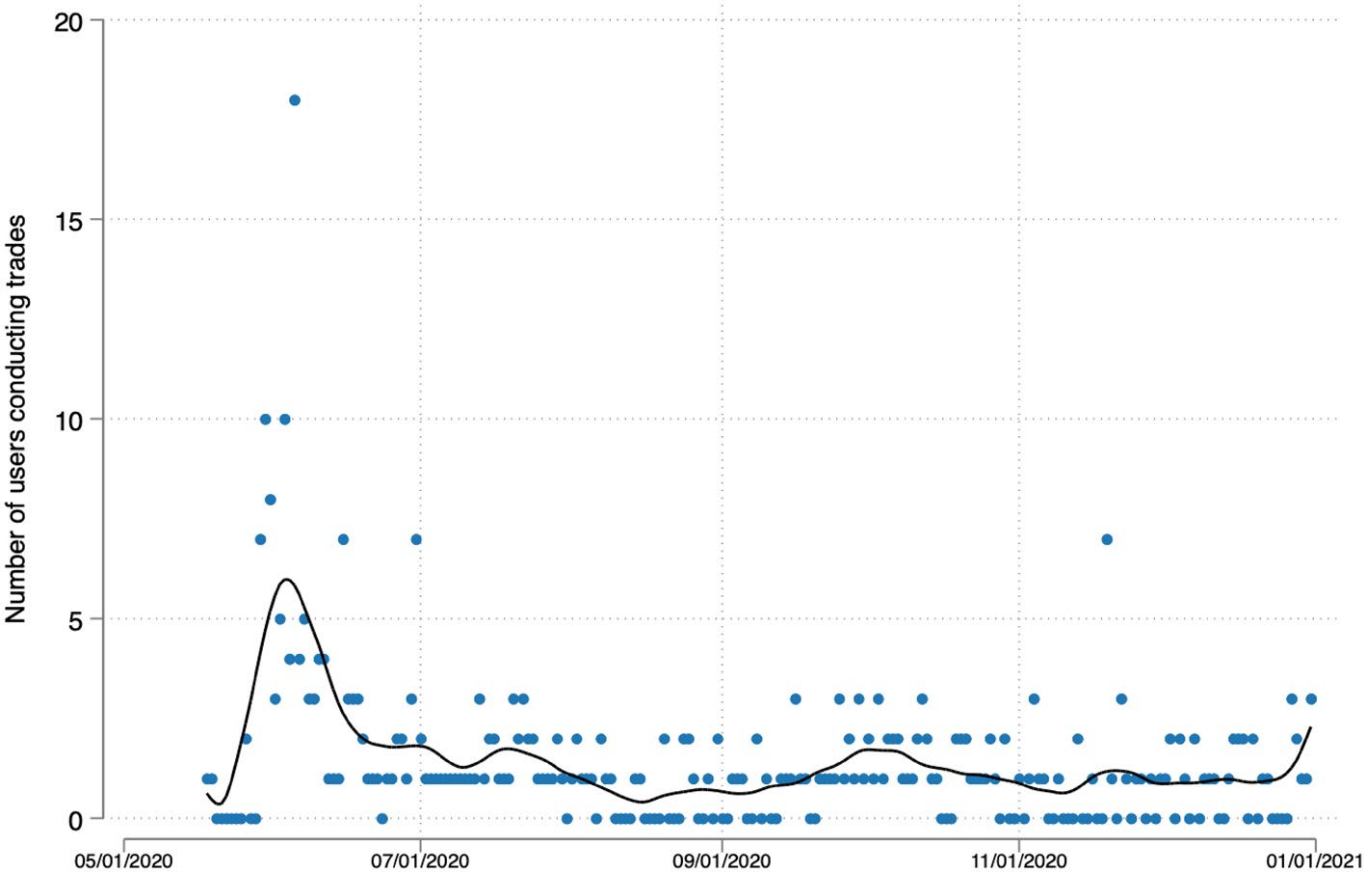
Number of participants conducting at least one trade. The dots show the number of participants conducting at least one trade for each day with the line showing the respective LOESS function with bandwith = 0.1

We sampled 425 experts and invited around 550 participants of previous prediction markets on elections in Spain and Switzerland.^11^ 164 individuals registered on the market, of which 97 conducted at least one trade. Figure 1 shows the number of participants that conducted at least one trade per day and Fig. 2 the total number of transactions performed. The trade activity peaked at the beginning of the project—the period from which we extract the forecasts analyzed here (see above). A second smaller peak can be observed in late September/early October when we reminded the participants.

Of the 97 individuals, 14 were experts, 35 were former participants on prediction markets for Switzerland, and 48 on prediction markets on Spanish politics. Consequently, 91% of the participants were from Spain (48.5%) or Switzerland (42.3%) with the rest from Austria, France, Germany, Poland, and the United Kingdom. As is typical for prediction markets, the large majority of participants were male (77%).

The laypeople outperformed the experts: While the laypeople increased their points during the game on average from 10′000 to 13′821, the experts ended up with a mean of 11′080 (the difference is not statistically significant at conventional levels, though). Since the sampling of the two groups has been very different, this does not mean that laypeople are as good or even better forecasters than experts. However, the fact that the laypeople fared similarly well as the experts is in line with the observation that highly dedicated laypeople might arrive in certain cases at least as good forecasts as experts. Experts might be overconfident (Tetlock, 2009, Tetlock and Gardner 2015), may—in cases like ours—have little advantage because they might not have access to much additional information, and tend to have more time constraints. One indication that time constraints might have played a role here is that on average experts have conducted fewer trades (5.9) than the laypeople (8.6). Independent of the reasons why the experts did not fare better than the laypeople, it suggests that in a prediction market laypeople can improve the prediction market’s forecast even for such a complex question as migration flows.

In theory, prediction markets work along a monetary logic and financial incentives are consequently assumed to be helpful to make sure that participants reveal their true expectations. However, as explained above, for ethical reasons participants were obliged to donate their wins to a humanitarian organization of their choice. (The average payout was 26.85 Euros (SD: 24.93) and a total of 2604.56 Euros was donated to five humanitarian organizations.) To maximize the payout for a good cause was probably a relevant motivation for some of the participants. Indeed, the fact that many would opt for one of four alternative organizations rather than the default organization that we offered for donation, suggests that these participants cared about this financial incentive (see part C in the Supplemental Material). We can also speculate that participants were motivated by social esteem since previous research has shown that on prediction markets without financial incentives this can be an important motivator (Qiu & Kumar, 2017). This is also why our prediction market provided a ranking on which each participant’s performance is visible for the other participants. Other motivations might be curiosity, supporting research or doing a personal favor to us. Based on our design it is impossible to know whether the prediction market would have been even more successful if stronger financial incentives could have been provided. This said, our mix of incentives did allow to recruit a sufficient number of actively trading participants and to create market efficiency that resulted in informed forecasts at least in the first couple of weeks.^12^

## Results

In what follows, we report the accuracy of the prediction market forecasts. In order to get a sense of the quality of the forecasts, we compare the average forecast on May 31 for the whole of 2020 with forecasts from simple standard time–series models based on data between 2008 and 2019 for the number of immigrants and between 2010 and 2019 for asylum applications.^13^

However, the two types of forecasts are not perfectly comparable: On the one hand, the prediction market had the advantage that it would generate forecasts for 2020 with knowledge regarding relevant events in the first months of the year for which the forecasts were made. On the other hand, participants could typically not rely on data for 2019, which is highly informative for 2020 and on which the time–series forecasts that were generated *ex post* heavily rely on.

The time–series forecasts that we use as a benchmark include the naïve forecast (i.e. the 2019 value), the drift (i.e. the 2019 value plus the trend), a forecast based on exponential smoothing, and one derived from a simple autoregressive integrated moving average (arima) model.^14^ Hence, we can think of prediction market forecasts that are far away from the actual value and further away than those from the different time–series forecasts as inaccurate and those that are close to the outcome and far from the other forecasts as relatively accurate.

Additionally, in order to judge the accuracy of the forecast we can assess whether the actual outcome falls within the 80% interval of our forecast. We make use of the narrow 80% confidence interval that is often used in forecasting since due to considerable uncertainty of ex-ante (relative to ex-post) predictions, this confidence interval is more informative than the larger 90% or 95% intervals (also Bijak, 2011).

**Fig. 3:**
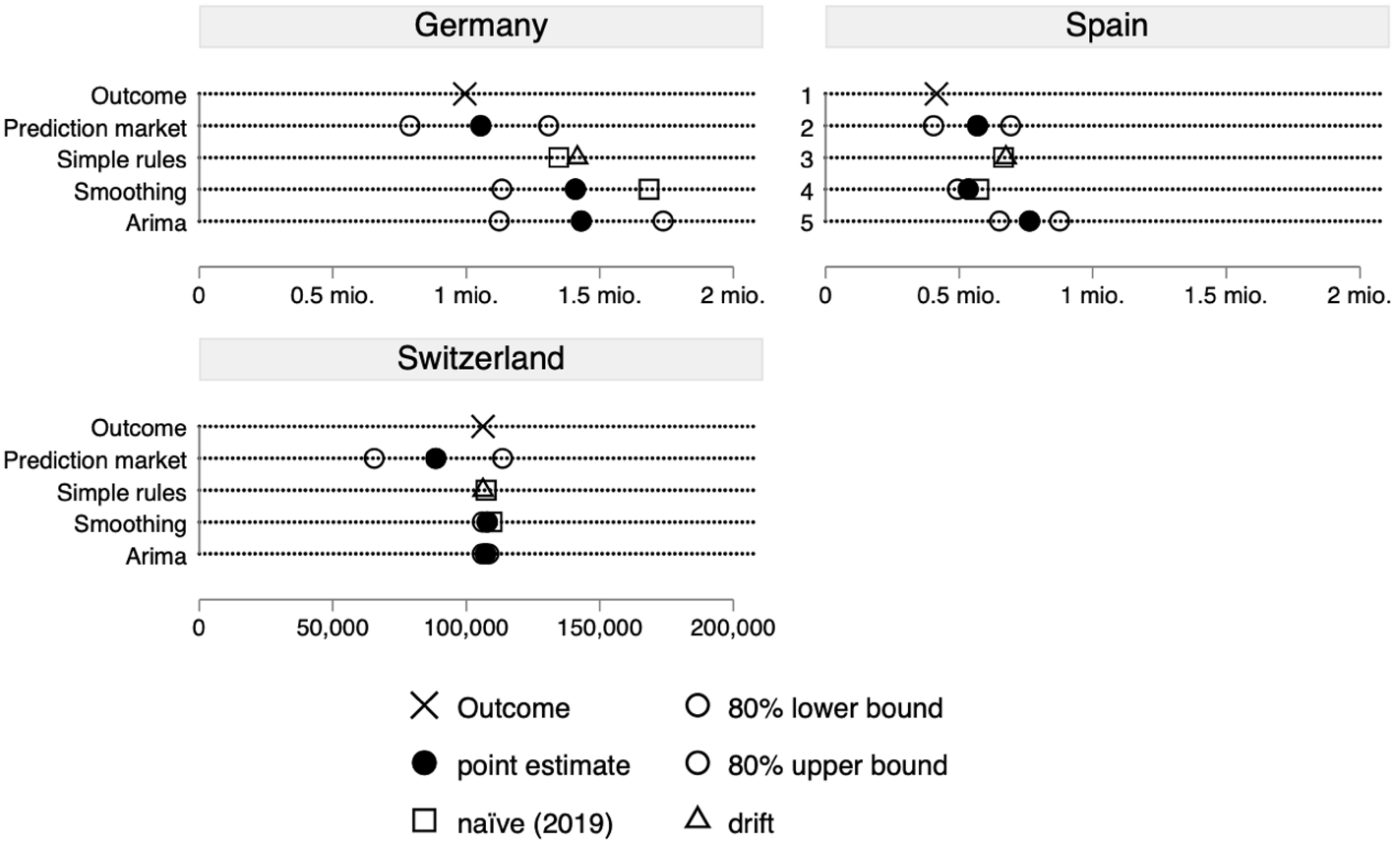
Forecasting accuracy for immigration in 2020. The outcome indicates the values for the year 2020, i.e. the value to be forecasted; The prediction market forecast are from May 31, 2020; The four different time–series forecasts have been conducted ex-post with all the data up to December 2019. The naïve forecast consists of the true value for 2019. For the data sources see the Supplemental Material part D

The first forecast to evaluate is the number of foreign immigrants in 2020. Figure 3 shows that for all three countries for which we generated a forecast, the outcome was within the 80% confidence interval. (Supplemental Material part F shows the same analysis including the time lines for the observed levels of immigration.) The forecasts came close to the actual outcomes in Germany and Spain. For Germany the forecasts were clearly more accurate than all time–series forecasts while for Spain this was true for three out of four time–series forecasts. Only the forecast based on exponential smoothing was closer to the real value than the prediction market forecast. The opposite is true for the case of Switzerland, where all time–series forecasts were very similar and clearly more accurate than the prediction market forecast. The reason is that the prediction market forecasted a decline in the number of non-native immigrants—such as was happening in Germany and Spain—while the time–series correctly forecasted a continuation of the very stable historical time–series in immigration to Switzerland. (The fact that the time–series forecasts are so similar and the confidence intervals so small is because for Switzerland the historical time–series is so stable.) Hence, with regards to total immigration of non-natives, the prediction forecast were closer to the real value in two out of three countries than the time–series forecasts, which we constructed as a benchmark.

**Fig. 4:**
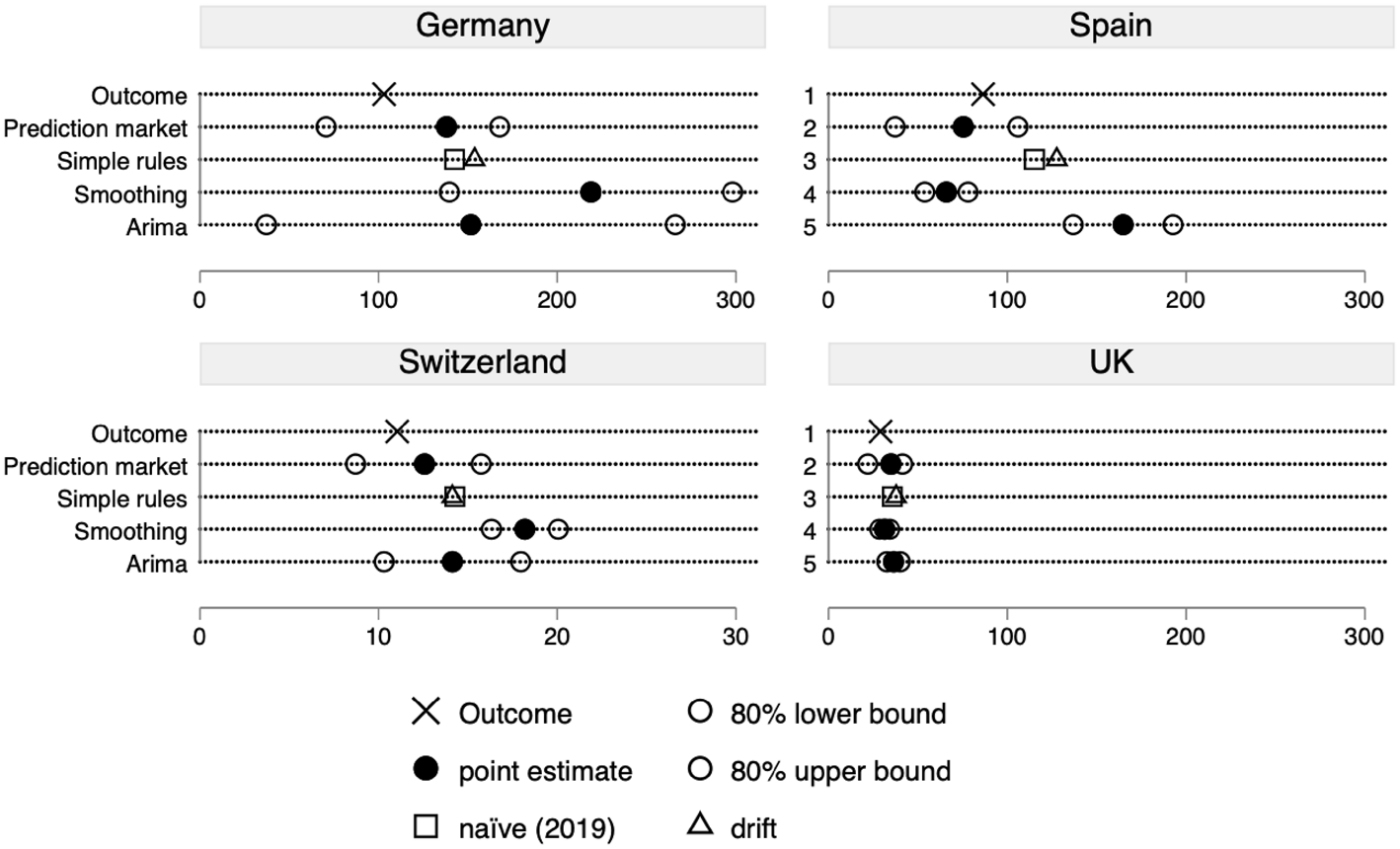
Forecasting accuracy for number of asylum applications in 2020. The outcome indicates the values for the year 2020, i.e. the value to be forecasted; The prediction market forecasts are from May 31, 2020; The four different time–series forecasts have been conducted ex-post with all the data up to December 2019. The naïve forecast consists of the true value for 2019. For the data sources see the Supplemental Material part D

With regards to the number of asylum seekers, we can evaluate the prediction market forecasts for all four countries. Figure 4 shows that again the real numbers were within the 80% confidence interval in all four cases. More interesting, the forecasts were typically closer to the time–series forecasts in all four countries. For Germany, Spain and Switzerland the prediction market forecasts were even more accurate than all time–series forecasts separately. For the UK only the forecast based on exponential smoothing was more accurate. In fact, the prediction market forecasts were way more accurate than any of the time–series forecasts. This is because while single time–series forecasts were pretty accurate for some countries, they tended to fail for others. For example, while the forecast based on exponential smoothing was highly accurate for the UK, it was far off for Germany and Switzerland. Hence, with regards to the number of asylum applications in 2020 the prediction market forecast would have been an improvement over simple time series forecasts.

We quantify the analysis of Figs. 3 and 4 in Table 1 by comparing the averages of the errors over the seven cases. We show for each type of forecast the Mean Absolute Error (MAE) for its simple interpretation as well as the Root Mean Square Error (RMSE) and the Mean Absolute Percentage Error (MAPE) that are very often used for their properties (see Hydman and Koehler 2006).^15^ The Table confirms that on average the prediction market has been more accurate than any of the time series forecasts. However, we should not overstate this result because one might still challenge that due to the late time point of the prediction market forecasts the comparison can be considered unfair. Hence, while our analysis seems to suggest that prediction markets have the potential for meaningful forecasts on immigration, we cannot conclude from these analyses that they are generally more accurate than time-series forecasts.

**Table 1:** Comparison of forecasting errors across methods

	Prediction market	Naïve	Drift	ARIMA	Exp. Smoothing
*Immigration*					
Mean absolute error (MAE)*	76	201	227	261	178
Root mean squared error (RMSE)*	95	249	286	322	248
Mean average percentage error (MAPE)	0.20	0.32	0.35	0.43	0.24
*Asylum applications*					
Mean absolute error (MAE)*	13	20	26	36	34
Root mean squared error (RMSE)*	19	25	33	59	46
Mean average percentage error (MAPE)	0.20	0.31	0.39	0.52	0.48

^*^In 1000 s

## Conclusion

In this article, we have introduced a new method for forecasting migration movements: prediction markets. While prediction markets are mainly unknown in migration studies, they are widely applied in the forecasting of election outcomes. The complexity of migration decision making and hence migration movements in the aggregate, make migration a particularly challenging topic to predict. We therefore adjusted the method of prediction markets to this constrained information environment of migration by two central aspects. First, we combine the wisdom of crowds with expert knowledge and second, we have argued for a probability market and the deduction of point estimates with measures for uncertainty through sequencing.

In order to assess the ability of prediction markets in general and the specific market design of this study, we applied the prediction market to forecasting immigration and asylum applications in four West European countries for 2020. As our analysis has revealed, the prediction market arrived at forecasts that were typically more accurate than predictions that would have been based on extrapolation. Hence, prediction markets can be considered a promising method for forecasting migration flows.

Yet, our application has not remained without limitations. First, our application has been small in scale covering only two different issues for four countries. Future research is needed extending in scale to verify the first results provided in this analysis. Second, the comparison of the prediction market with the time series forecasts in this analysis has been far from ideal. The reason is that the prediction market forecasts were based on later data points than the time series forecasts. Furthermore, an investigation into the rapidly evolving machine learning methodologies and the function of 'nowcasting' techniques within this context would be a valuable addition to the existing literature. Hence, future studies should strive at better comparability in forecasts across methods for example in the form of a forecasting tournament. Finally, we were less successful than expected in recruiting experts for the prediction market. In order to increase the number of experts that participate, we would need to improve the means to convince experts of its value and—if possible—provide more (non-monetary) incentives to it (such as participation in joint publications with the generated data). This would not only increase the forecasting accuracy of the market but also allow it to fully exploit its advantages by generating an extended time–series of updated forecasts. Additionally, it would be an asset to sample stronger on migrants as another group of migration experts with diverse and often private information.

We are convinced that—following additional verification—prediction markets will be a valuable complement to more traditional methods in migration forecasting be it for state agencies, non-governmental organisations (NGOs), or society at large. Its advantages of rapid updating in limited and complex information settings will be an asset here. For example, in the context of emerging crisis such as war or environmental disaster the quickly updating prediction market can provide valuable information that can challenge and/or complement individual expert opinions. However, a prediction market on migration presumably will not arise naturally and it is a major challenge to convince potential funders such as academic and state agencies to provide the means needed.

## Supplementary Information

Additional file 1.

## Data Availability

The data and replication code are available at Harvard Dataverse: https://doi.org/10.7910/DVN/YW4QDF.

## Footnotes

1 Although prediction markets are a powerful method for forecasting, prediction markets have not been successfully commercialized on a large scale. Commercial prediction market providers earn from transaction fees on participants trades and/or other fees. However, potential prediction market participants are usually not ready to pay these fees except for highly salient (e.g. US presidential elections) and/or highly entertaining issues (e.g. sports) (Snowberg, Wolfers, and Zitzewitz 2013). Similarly, there is usually not enough demand for economic forecasts because—assuming a continuation of the historical trend in stock market growth—trading on the stock market comes with a higher expected value than trading on prediction markets on which average return to investments is constant (Silver 2012). For these reasons, there is to our knowledge currently no commercial prediction market on which issues regarding migration flows are forecasted. Consequently, in order to provide a prediction market on which questions regarding migration movements are forecasted the organizer must change the incentive structure by providing financial incentives to the potential participants (see below).

2 The purpose of registration data is to collect knowledge about who is currently officially in the country, i.e., receives benefits, is eligible for work, etc. Our research interest is, however, that of numbers regarding immigration and emigration.

3 A specific advantage of prediction markets relative to other markets is that since the market’s time horizon is typically rather short, even in the case of inefficient markets, the probability of ‘speculation bubbles’ is small.

4 Utility maximizers in the context of stock markets are individuals that maximize the expected profit. They are for example different from “satisficers” who only maximize their utility to a certain value. The utility function whose expected value is maximized is linear for a risk neutral agent. A utility maximizer is risk neutral if the expected utility of her profit is simply equal to the expectation of a linear function of profit.

5 This is of course only true if the trader has enough liquidity to conduct the trades and no other contracts deviate in the eyes of the trader more from the probability of the event taking place.

6 We use standard linear interpolation. The value y at x is found by finding the closest points (x_0_, y_0_) and (x_1_, y_1_), such that x_0_ < x and x_1_ > x where y_0_ and y_1_ are observed. We calculate $\text{y}=\frac{{y}_{1}-{y}_{0}}{{x}_{1}-{x}_{0}}(x-{x}_{0})+{y}_{0}$

7 It is sometimes argued that prediction markets have a comparative advantage for forecasts in the «long run» (Berg, Nelson, and Rietz 2008). However, this is only true to the degree that the traders are not strongly discounting for payout of trades with regards to events in the distant future relative to those in the near future. Otherwise, markets for events in the distant future suffer from inefficient markets (Page and Clemen 2013). In order to limit this negative effect of the discount rate we only offered ‘contracts’ that would settle on the same date in the not so near future (eight months).

8 For LMSR, the cost function is: C = b * ln(Eq. ^1^^/b^ + Eq. ^2^^/b^), where “ln” is the natural logarithm function, “e” is the constant e = 2.718…, and “b” is a parameter that the market maker must choose. Due to the LMSR’s logarithmic function, it becomes increasingly expensive to push the price further down from the midpoint towards the minimum and increasingly expensive to push the price further up from the midpoint towards the maximum. In a context of cash constraints, this could lead to the overpricing of contracts for which the expected final price is low and the underpricing of contracts for which the final price is expected to be high (Arnesen & Strijbis 2015).

9 We understand “nowcasts” as the prediction of the very recent past, the present, and the very near future event whose outcome is unknown. For practical purposes, these predictions on past and current values—a form of “monitoring”—can be just as relevant as forecasts for future time points.

10 Among the laypeople, we could draw on participants who have successfully participated in a previous prediction market on other topics like recent elections in Spain and Switzerland. These participants are also highly valuable because they understand the logic of the prediction market well and can exploit inefficiencies in the market, for example, through arbitrage gains.

11 We counted 554 Emails sent to 223 participants of a prediction market on Spanish elections and 331 to participants of prediction markets on Swiss elections and direct democratic votes.

12 However, together with the limited number of participants that we recruited the incentives were not strong enough to exploit a crucial potential asset of the prediction market, which is the generation of time–series of quickly updated forecasts.

13 An alternative would be to compare our predictions with nowcasts. Unfortunately, for the countries and time period of our analysis no nowcasts were available.”.

14 In order to make comparisons based on ex post fitting as meaningful as possible, we opted for standard specifications: For the smoothing we chose as weighting parameter 0.4 and estimated an arima model on differenced data (d = 1) with one lagged data point (p = 1) and one lagged error for the moving average part (q = 1), i.e. an arima (1,1,1) specification. Confidence intervals were calculated with bootstrapping with three–year periods specified as clusters (“blocs”). Supplemental Material part E shows results from alternative arima specifications.

15 Reasons are that the RMSE is less sensitive to outliers than the MAE and the MAPE is scale-independent.
