# Supplementary material for: Forecasting migration movements using prediction markets
Source: Comp Migr Stud. 2024 Oct 9;12(1):45. doi: 10.1186/s40878-024-00404-0 (PMC11464559; doi:10.1186/s40878-024-00404-0)

# **Appendix**

## **A. Study Design**

Figure A.1: Timeline Study Implementation


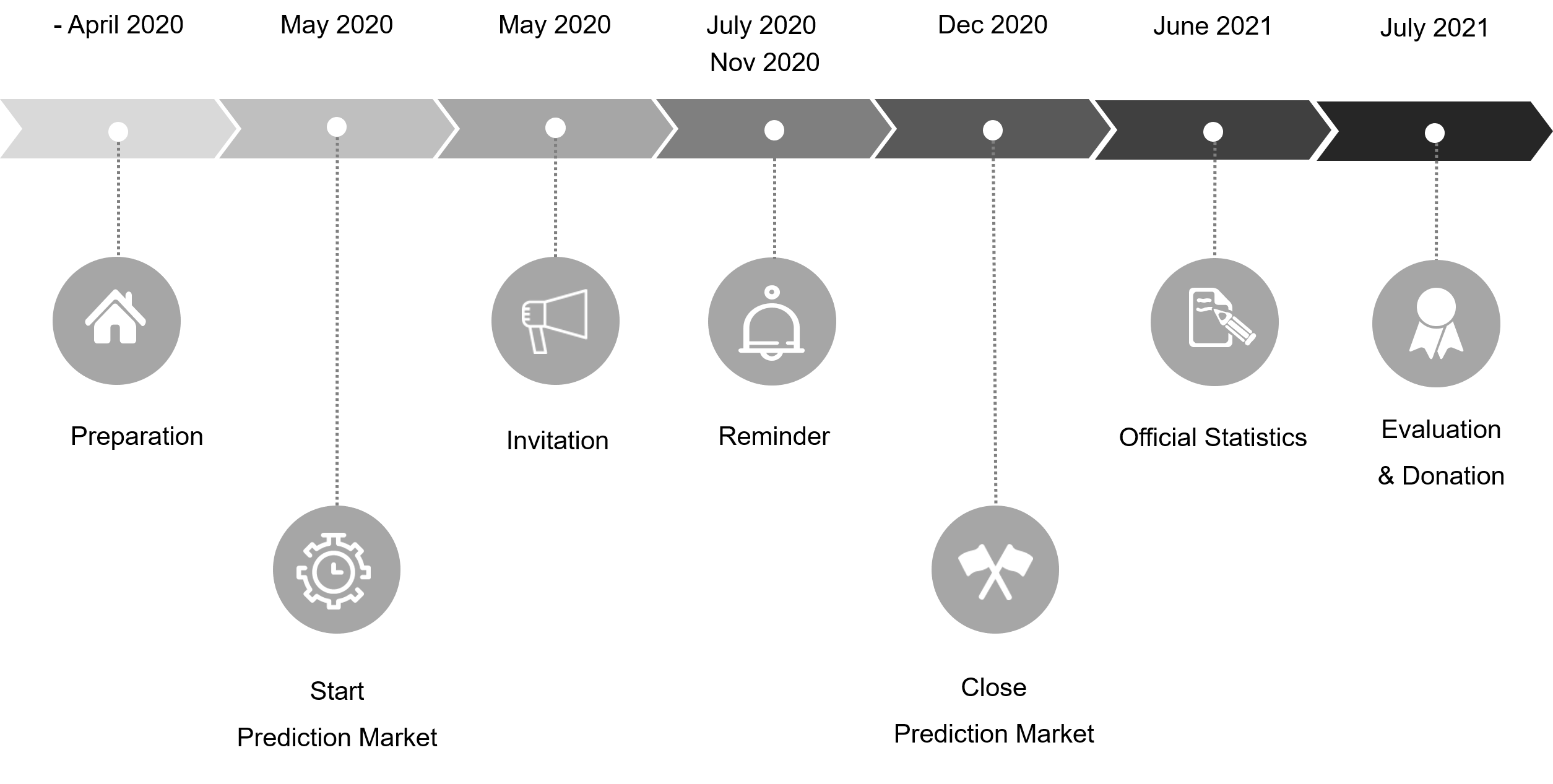


**B. Study Implementation**

Figure B.1: Picture of a contract–set in the prediction market on migration flows


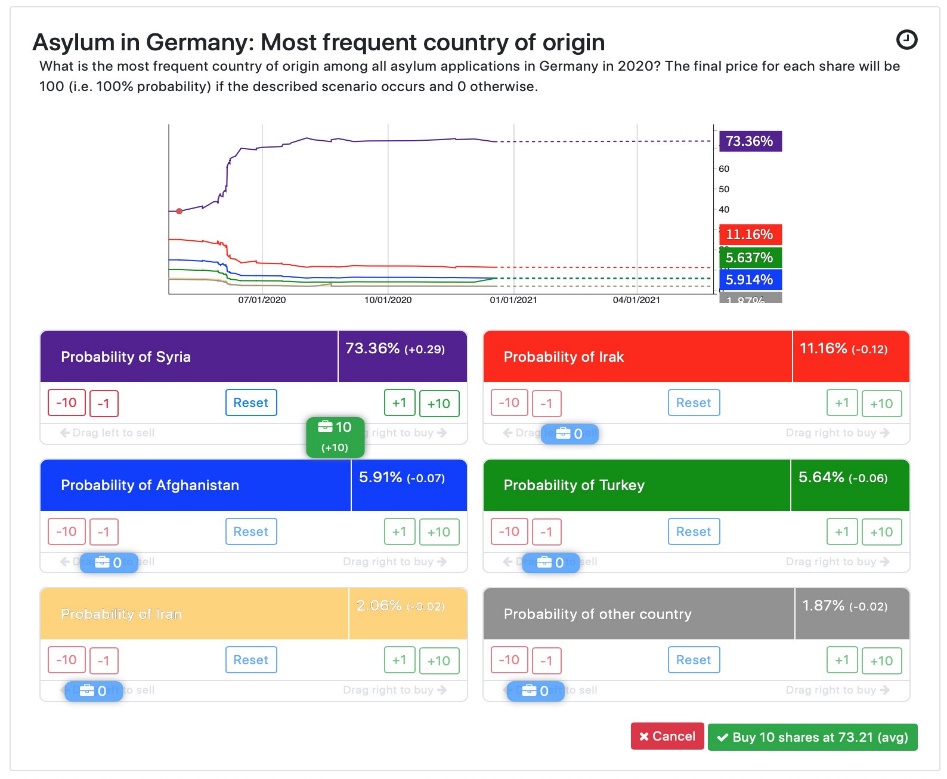


**C. Participant information**

The following tables give some basic descriptives on the participants and the payouts.

Table C.1: Participants by expert status and gender

| Expert | Female | Male | Missing | Total |
| --- | --- | --- | --- | --- |
| Yes | 4 | 10 | 0 | 14 |
| % | 28.57 | 71.43 | 0 | 100 |
| No | 15 | 65 | 3 | 83 |
| % | 18.07 | 78.31 | 3.61 | 100 |
| Total | 19 | 75 | 3 | 97 |
| % | 19.59 | 77.32 | 3.09 | 100 |
| Note: Experts are professionals in research, NGOs or public administration. Gender classified based on names. | | | | |

Table C.2: Participants by country of residence

| Country | Frequency | Percent (%) |
| --- | --- | --- |
| Austria | 1 | 1.03 |
| France | 1 | 1.03 |
| Germany | 4 | 4.12 |
| Poland | 1 | 1.03 |
| Spain | 47 | 48.45 |
| Sweden | 1 | 1.03 |
| Switzerland | 41 | 42.27 |
| United Kingdom | 1 | 1.03 |
| Total | 97 | 100 |
| Note: Country measures the country of the experts’ institution or the country to which the Email address can be related to (usually official University emails). | | |

Table C.3: Payouts to humanitarian organizations

| Organization | Euros |
| --- | --- |
| Amnesty | 69.04 |
| Caritas | 58.36 |
| MSF | 188.42 |
| Red Cross | 61.95 |
| UNHCR (default option) | 2226.79 |
| Total | 2604.56 |

**D. Data sources**

General comment: In order to give the participants on the prediction market a benchmark from which they could form expectations even with limited information, we indicated them past values on immigration and asylum application numbers from the year 2018 or if available (provisional) data for the year 2019. Often national institutions (e.g. ministries of immigration) published the accounts first, which is why we tended to rely on their data. The values of this data can deviate to some extent of the standardized European accounts of Eurostat. Below we provide the data and sources that we used to calculate the time series forecasts.

Table D.1: Non-native immigrants, 2008-2020

| Year | Spain | Germany | Switzerland |
| --- | --- | --- | --- |
| 2020 | 415150 | 994819 | 106231 |
| 2019 | 666022 | 1345943 | 107387 |
| 2018 | 559998 | 1383581 | 106986 |
| 2017 | 453950 | 1384018 | 105040 |
| 2016 | 352174 | 1719075 | 109623 |
| 2015 | 290005 | 2016241 | 112319 |
| 2014 | 264485 | 1342529 | 110131 |
| 2013 | 248350 | 1108068 | 119366 |
| 2012 | 272489 | 965908 | 112346 |
| 2011 | 335893 | 841695 | 113141 |
| 2010 | 330286 | 683530 | 106299 |
| 2009 | 365367 | 606314 | 104010 |
| 2008 | 567372 | 573815 | 118629 |

Immigration was defined as the number of non-native immigrants in the year 2020. Data was taken from the following sources:

- Spain: Flujo de inmigración procedente del extranjero por año, país de origen y nacionalidad (española/extranjera): <https://www.ine.es/jaxiT3/Datos.htm?t=24295>
- Germany: Wanderungen zwischen Deutschland und dem Ausland 1991 bis 2020: <https://www.destatis.de/DE/Themen/Gesellschaft-Umwelt/Bevoelkerung/Wanderungen/Tabellen/wanderungen-alle.html>
- Switzerland: Zu- und Abnahme ausländische Wohnbevölkerung: Effektive Einwanderung: <https://www.sem.admin.ch/sem/de/home/publiservice/statistik/auslaenderstatistik/archiv.html>

In the case of Germany, the data is based on a total survey based on administrative data (secondary data). It is based on the registrations and deregistrations recorded by the registration offices of the federal states in accordance with registration regulations. For more details see: Statistisches Bundesamt (2023): "Wanderungen (Binnenwanderung, Außenwanderung, Gesamtwanderung)". Url (as of November 27, 2023): <https://www.destatis.de/DE/Methoden/Qualitaet/Qualitaetsberichte/Bevoelkerung/wanderungsstatistik-2022.pdf?__blob=publicationFile>

Also in the Swiss case, the count is based on register data. The sum of immigration is made up of effective immigration, increase due to change of status, and transfers from the asylum sector. The sum of emigration is made up of effective emigration and decrease due to change of status. For further details see: Staatssekretariat für Migration SEM (2015): "Wichtige Hinweise zu Änderungen in den Ausländer-Statistiken des Staatssekretariats für Migration SEM". Url (as of November 27, 2023): https://www.sem.admin.ch/sem/de/home/publiservice/statistik/auslaenderstatistik/archiv/2022/02.html

Table D.2: First time asylum applications in four countries, 2010-2020

| Year | Switzerland | Germany | Spain | UK |
| --- | --- | --- | --- | --- |
| 2010 | 15567 | 41245 | 2550 | 17916 |
| 2011 | 22551 | 45680 | 2970 | 19865 |
| 2012 | 28631 | 64410 | 2350 | 21843 |
| 2013 | 21465 | 109375 | 4285 | 23584 |
| 2014 | 23765 | 172945 | 5460 | 25033 |
| 2015 | 39523 | 441805 | 14600 | 32733 |
| 2016 | 27207 | 722270 | 15570 | 30747 |
| 2017 | 18088 | 198255 | 33035 | 26547 |
| 2018 | 15255 | 161885 | 52730 | 29504 |
| 2019 | 14269 | 142450 | 115175 | 35737 |
| 2020 | 11041 | 102525 | 86380 | 29456 |

The number of asylum applications was defined as the number of first asylum applications in 2020. Data was taken from the following sources:

- UK: Home Office. 2021. "Immigration Statistics year ending December 2020" Asylum and Resettlement - Summary tables. Published: 25 February 2021. <https://www.gov.uk/government/statistics/immigration-statistics-year-ending-december-2020.>

### Germany and Spain: Eurostat. 2021. “Asylum and first time asylum applicants - annual aggregated data (rounded). Asylum and first time asylum applicants - annual aggregated data (rounded).”

### <https://ec.europa.eu/eurostat/databrowser/view/tps00191/default/table?lang=en>

### Switzerland: Staatssekretariat für Migration SEM. Asylstatistik 2020, p.14. Published on February 1, 2021.

### <https://www.sem.admin.ch/dam/sem/de/data/publiservice/statistik/asylstatistik/2020/stat-jahr-2020-kommentar.pdf.download.pdf/stat-jahr-2020-kommentar-d.pdf>

For the five most frequent countries of origins of the first time asylum applications we relied on data from Eurostat:

### Eurostat. 2020. «Table 1: Five main citizenships of first-time asylum applicants (non-EU citizens), 2020 (number, rounded figures)». Published on March 23, 2021. <https://ec.europa.eu/eurostat/statistics-explained/index.php?title=File:Table_1_Five_main_citizenships_of_first-time_asylum_applicants_(non-EU_citizens),_2020_(number,_rounded_figures)_v2.png>

**E. Alternative specifications for arima models**

In the main text, we have compared the prediction market forecasts with time series models. One of these models consisted of an arima(1,1,1) model. Below, we provide a table with the forecasts from alternative specifications both for the number of immigrants (Figure 3 in the main text) and number of first time asylum applications (Figure 4 in the main text). In our search for alternative specifications, we inspected correlograms and partial correlograms for first and second differenced data. For immigration, none of the (partial) correlograms indicated to increase p or d beyond 1. For asylum applications, the partial correlograms would have indicated that p=2 might be more appropriate than p=1. However, due to the very short time series arima(2,1,1) could not be estimated for asylum applications.

Table E.1: Forecasts of immigration based on alternative arima specifications

| arima | Switzerland | Germany | Spain |
| --- | --- | --- | --- |
| p=1, d=1, q=1 | 107151 | 1430052 | 763316 |
| p=2, d=1, q=1 | 107271 | 1401467 | 763227 |
| p=1, d=2, q=1 | 107134 | na* | 803676 |
| p=1, d=1, q=2 | 108110 | na* | 740147 |
| Note: Estimations conducted with the arima command in Stata 17; *Estimation failed | | | |

Table E.2: Forecasts of immigration based on alternative arima specifications

| arima | Switzerland | Germany | Spain | United Kingdom |
| --- | --- | --- | --- | --- |
| p=1, d=1, q=1 | 14130 | 151597 | 164791 | 164791 |
| p=1, d=2, q=1 | 7016 | 52020 | 171561 | 38817 |
| p=1, d=1, q=2 | 19201 | 246186 | 180499 | 37430 |
| Note: Estimations conducted with the arima command in Stata 17 | | | | |

**F. Forecasts of most important country of origin of first–time asylum applicants**

We have also analyzed the forecasting accuracy for the country of origin with most asylum applicants in 2020. Because the value to be forecasted is a dummy variable, it is more complicated to provide a meaningful evaluation of the forecast. Hence, to assess the forecasting accuracy we do not only assess if the country of origin that was forecasted the highest probability of being the most frequent one actually was. Instead, we also compare whether the rank order of the probabilities correlates with the rank order of the number of asylum applications. Taking this information to evaluate our forecasts is intuitive because the actual number of applications by country of origin is most likely strongly related to the probability that this country of origin would be the most frequent. For example, if the number of applications from the two most frequent countries of origin are very similar, it is also very likely that the probability for each of them being the most frequent is about the same.

Figure F.1: Forecasting accuracy for the origin of asylum applicants in 2020


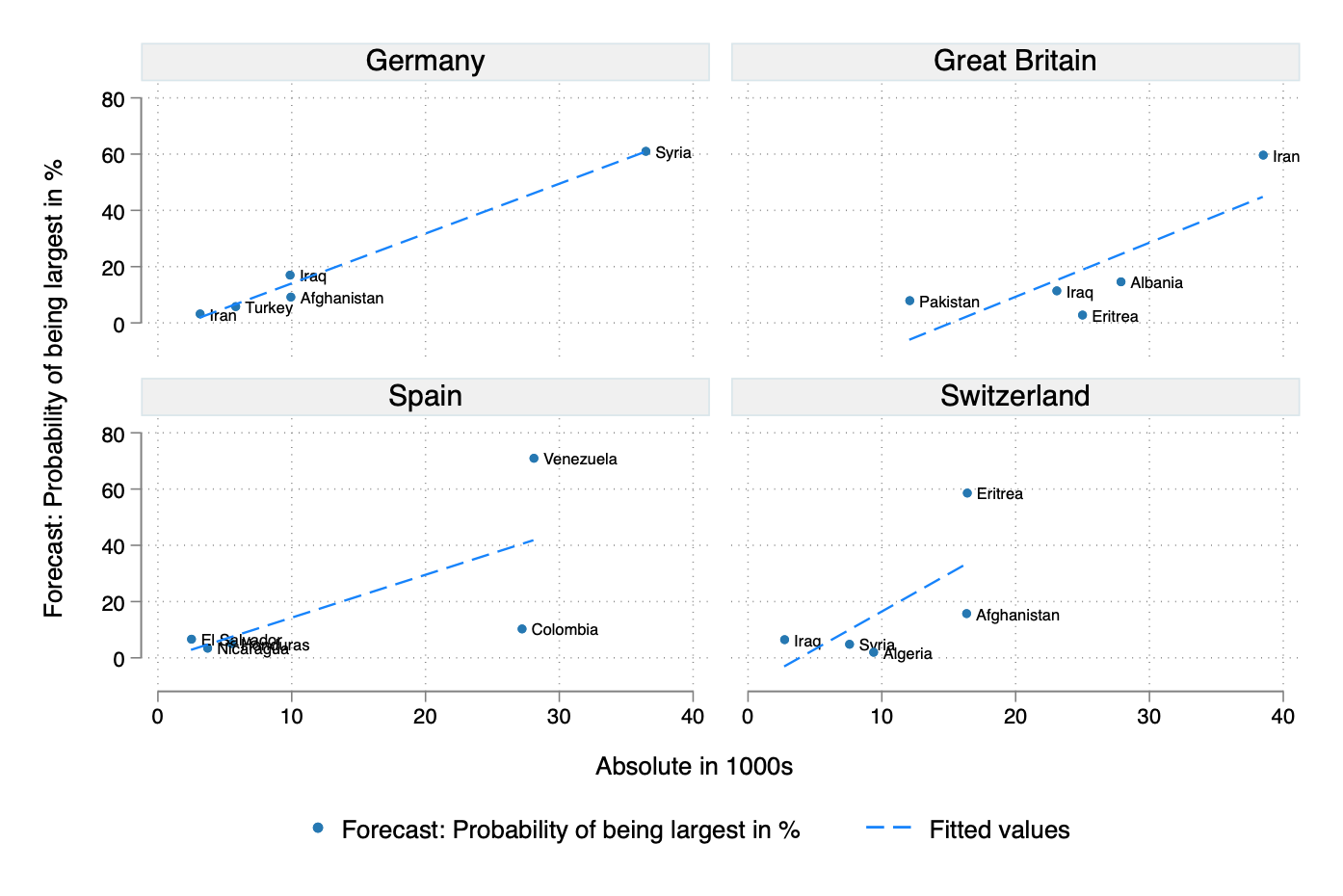


Notes: The blue lines are OLS regression fit lines describing the co-variance between the actual number of asylum applicants (x-scale) and the forecasted probability that the country of origin would be the most frequent source of asylum applications (y-scale). A steep line indicates that the actual number of applications is positively related to the forecasted probability of being the most frequent country of origin. For the data sources see the Supplemental Material part D.

In order to estimate the probability of the country of origin being the most frequent source of asylum applications, participants might have extrapolated from patterns in the past. In this regard, it is important to know that depending on the country the patterns were more or less clear. In Germany, for example, the by far largest group of first–time asylum applicants were Syrian refugees since 2014. In Switzerland, in contrast, the largest group varied depending on the year. Syrians or Eritreans were the largest group while the refugees from Afghanistan usually followed as the second or third largest. In Spain, in turn, clearly the largest group over the last four years were the Venezuelans. In the UK, the pattern is less clear although between 2017 and 2019 the largest number of first–time asylum seekers always came from Iran.

Figure E.1 shows that in all four countries, the highest probability was attached to those countries of origin that turned out to be the most frequent sending states. Hence, strictly speaking, for all four countries, the forecast was correct. We can also see that in the two cases where these numbers turned out to be relatively close, the second most frequent country of origin received the second highest probability. Hence, also in these more complicated cases, the forecasts were correct. However, one might want to argue that the probabilities of these second most frequent countries of origins (Colombia in Spain and Afghanistan in the UK) that were clearly below 50% are underestimations of the actual probabilities at the time and hence that in these cases the forecasts were somewhat off. It is impossible to know whether this is true or the probabilities of the forecasts were fully correct. In sum, however, it is evident that by correctly forecasting in all cases which would be the most frequent country of origin and a positive correlation between the probability of being the most frequent and the actual numbers, the prediction market forecasts can be considered highly accurate.

**G. Replication of Figures 3 and 4 as time line graphs**

In the subsequent graphs we show the same forecasts as in Figures 3 and 4. In contrast to Figure 3 and 4, we show however also the observed values for immigration and asylum applications for the years before 2020. Also, for reasons of presentation we do not show confidence intervals.

Figure G.1: Forecasting accuracy for immigration in 2020

1. Switzerland


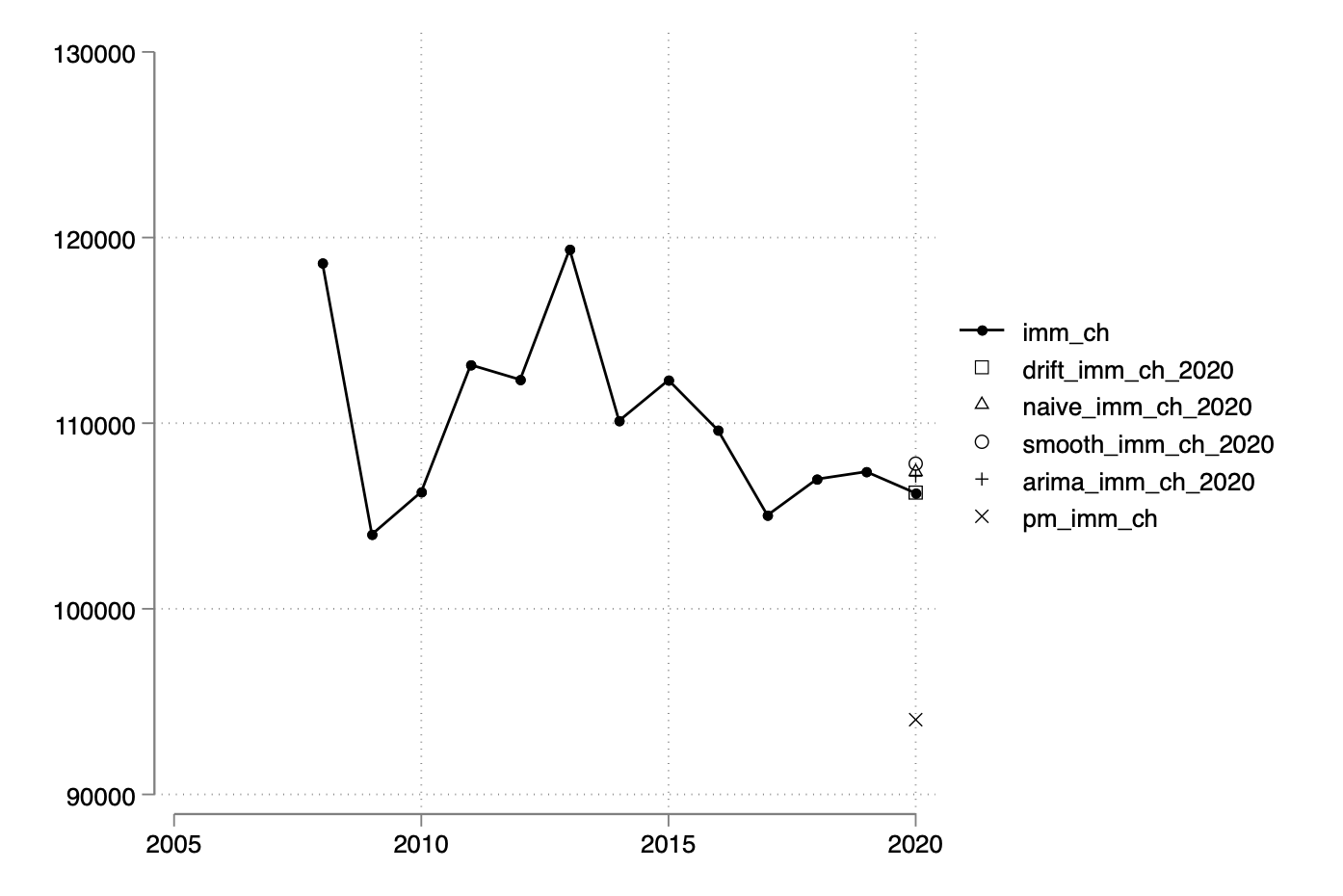


1. Germany


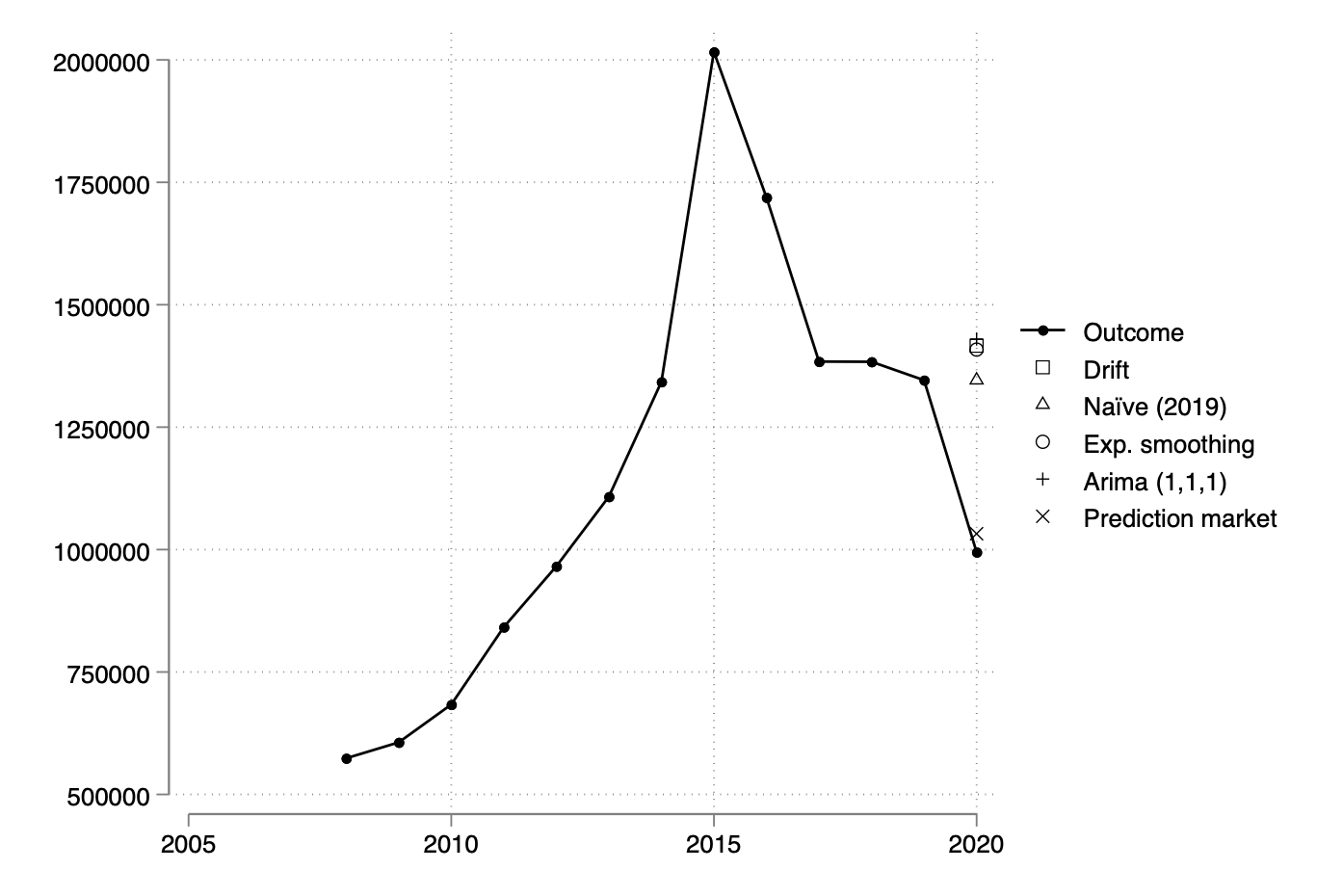


1. Spain


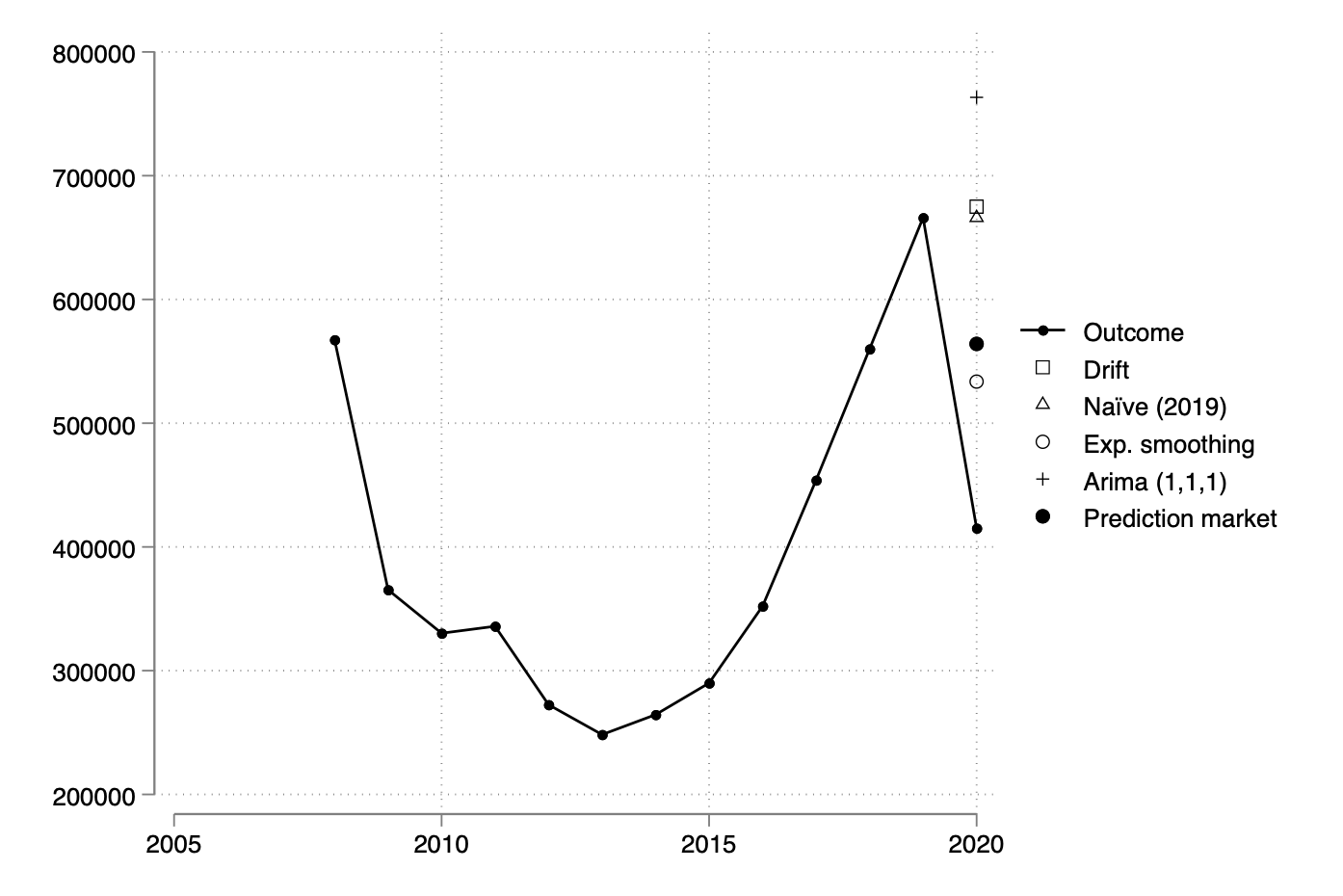


Figure G.2: Forecasting accuracy for number of asylum applications in 2020

1. Switzerland


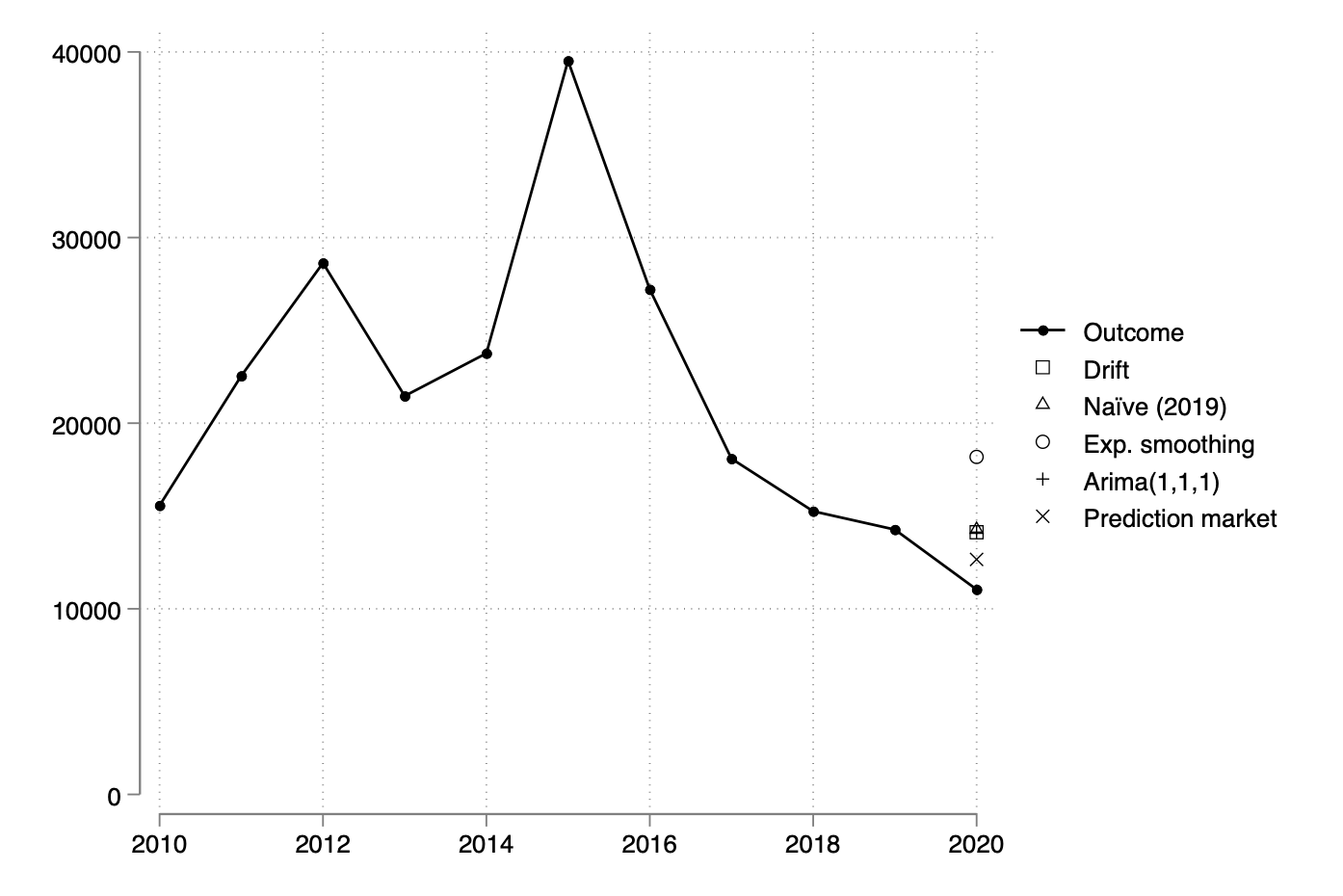


1. Germany


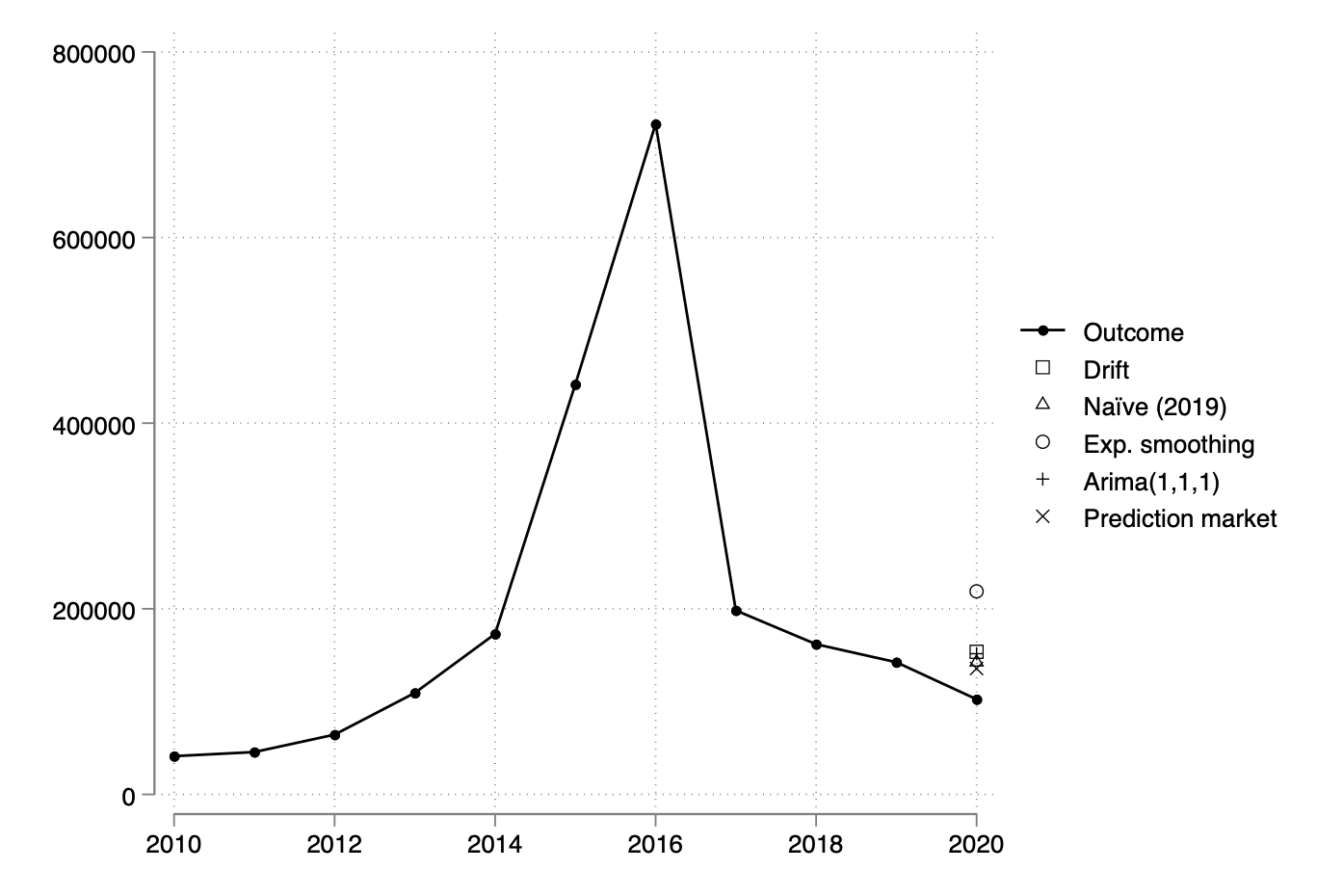


1. Spain


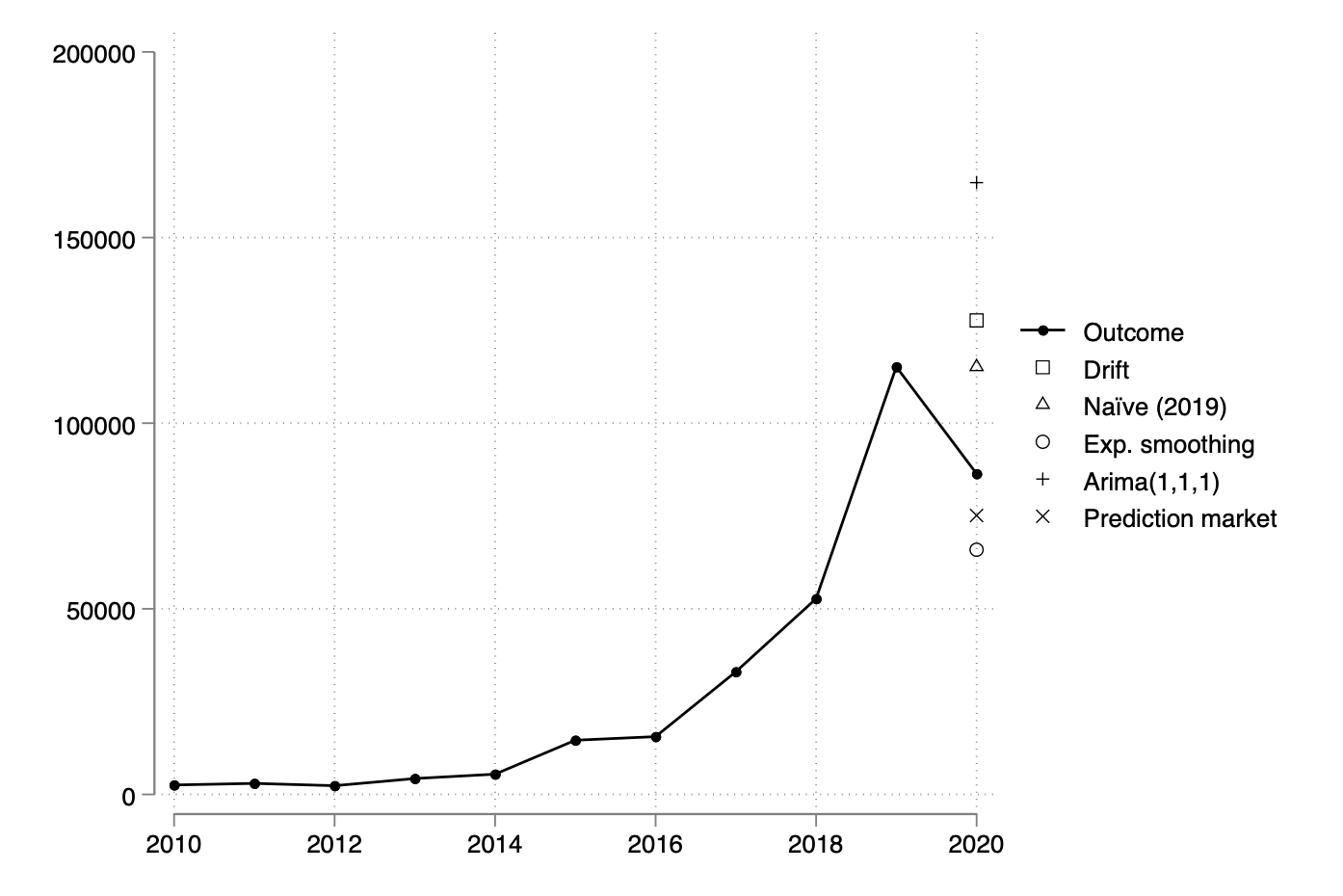


1. United Kingdom


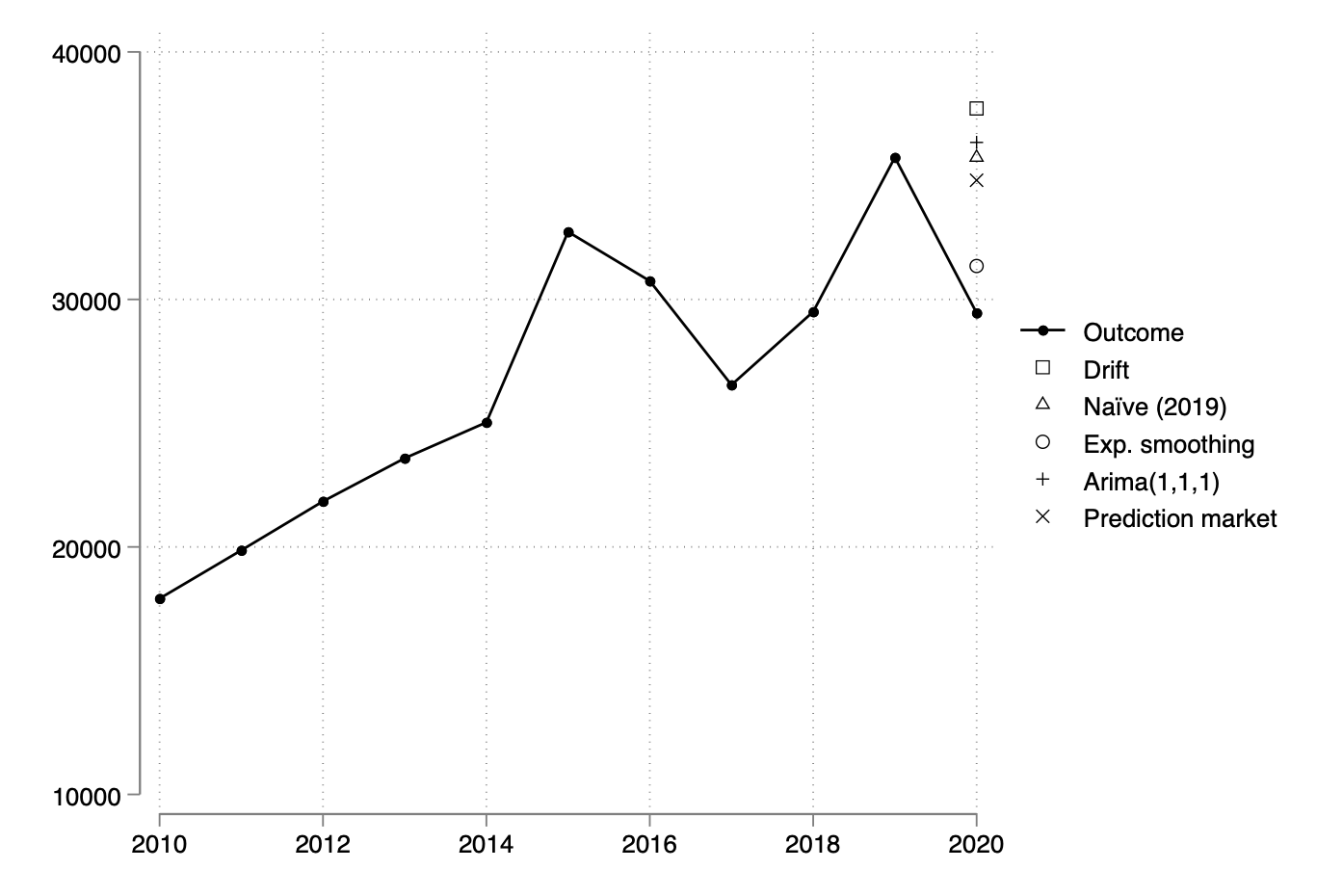

Supplement: Supplementary file 1 — Additional file 1. [file 40878_2024_404_MOESM1_ESM.docx]
